# Severe hypokalemic paralysis and rhabdomyolysis occurring after binge eating in a young bodybuilder: Case report: Erratum

**DOI:** 10.1097/MD.0000000000008478

**Published:** 2017-10-27

In the article, “Severe hypokalemic paralysis and rhabdomyolysis occurring after binge eating in a young bodybuilder: Case report”,^[1]^ which appeared in Volume 96, Issue 40 of *Medicine*, Dr. Ha Nee Jang's name appeared incorrectly as Ha Nee Chang.
